# Impact of the Covid-19 pandemic on the performance of machine learning algorithms for predicting perioperative mortality

**DOI:** 10.1186/s12911-023-02151-1

**Published:** 2023-04-12

**Authors:** D. I. Andonov, B. Ulm, M. Graessner, A. Podtschaske, M. Blobner, B. Jungwirth, S. M. Kagerbauer

**Affiliations:** 1 Department of Anaesthesiology and Intensive Care Medicine, School of Medicine, Technical University of Munich, Munich, Germany; 2 Department of Anaesthesiology and Intensive Care Medicine, School of Medicine, University Hospital Ulm, University of Ulm, Albert-Einstein-Allee 23, Ulm, 89081 Germany

**Keywords:** Machine learning, Model degradation, Covariate shift, Covid-19

## Abstract

**Background:**

Machine-learning models are susceptible to external influences which can result in performance deterioration. The aim of our study was to elucidate the impact of a sudden shift in covariates, like the one caused by the Covid-19 pandemic, on model performance.

**Methods:**

After ethical approval and registration in Clinical Trials (NCT04092933, initial release 17/09/2019), we developed different models for the prediction of perioperative mortality based on preoperative data: one for the pre-pandemic data period until March 2020, one including data before the pandemic and from the first wave until May 2020, and one that covers the complete period before and during the pandemic until October 2021. We applied XGBoost as well as a Deep Learning neural network (DL). Performance metrics of each model during the different pandemic phases were determined, and XGBoost models were analysed for changes in feature importance.

**Results:**

XGBoost and DL provided similar performance on the pre-pandemic data with respect to area under receiver operating characteristic (AUROC, 0.951 vs. 0.942) and area under precision-recall curve (AUPR, 0.144 vs. 0.187). Validation in patient cohorts of the different pandemic waves showed high fluctuations in performance from both AUROC and AUPR for DL, whereas the XGBoost models seemed more stable. Change in variable frequencies with onset of the pandemic were visible in age, ASA score, and the higher proportion of emergency operations, among others. Age consistently showed the highest information gain. Models based on pre-pandemic data performed worse during the first pandemic wave (AUROC 0.914 for XGBoost and DL) whereas models augmented with data from the first wave lacked performance after the first wave (AUROC 0.907 for XGBoost and 0.747 for DL). The deterioration was also visible in AUPR, which worsened by over 50% in both XGBoost and DL in the first phase after re-training.

**Conclusions:**

A sudden shift in data impacts model performance. Re-training the model with updated data may cause degradation in predictive accuracy if the changes are only transient. Too early re-training should therefore be avoided, and close model surveillance is necessary.

**Supplementary Information:**

The online version contains supplementary material available at 10.1186/s12911-023-02151-1.

## Introduction

In the spring of 2020, the Covid-19 pandemic rapidly changed clinical routines in our hospitals with staff redeployment and elective procedures postponed due to increased demand for ICU beds. The surgical spectrum shifted to emergencies. During the lockdown, the number of trauma cases declined, and some centres reported fewer surgeries outside normal operating hours [1]. Patients who still underwent surgery were, on average, older and sicker than before the pandemic. Such changes in surgical spectrum and patient characteristics can lead to shifts in feature importance and affect the performance of machine learning models [2]. In the medical field, there are few studies on the degradation of predictive models due to evolving data [3]. In general, it is known that models performing well initially can degrade as the data changes in course of the progress [4]. This so-called “data drift” is gradual in most cases, however, external events can cause a sudden change in feature distribution, i.e., a covariate shift. Another issue is the incidence of the endpoint which may also be affected if, for example, mortality risk increases [5]. To handle covariate shifts, some researchers suggest that past data should be “forgotten” or down-weighted [4]. The question of whether and at what intervals a model needs to be re-trained is difficult to answer. Many models used in economics experience automatic updates at specific time intervals. However, it is not clear whether this approach is also the right one for models in the clinical setting [6]. This is all the more important as predictive models in surgical medicine have their practical value especially in times of rapidly approaching resource scarcity. An important aspect in this context is, for example, to support responsible and at the same time efficient operating room and intensive care unit (ICU) bed planning based on individual patient risk. The aim of our study was to analyse the predictive quality of machine-learning models in different time periods of the pandemic and to identify whether re-training with updated data helps to make predictions more reliable.

To address this question, we developed machine learning algorithms to predict perioperative mortality based on preoperatively available data. We did this by creating XGBoost models and Deep Learning neural networks (DL) for three different time periods: one with pre-pandemic data, one with pre-pandemic and first-wave data through May 2020, and one with data from the complete period before and during the pandemic until October 2021. We compared the performance metrics of each model during the different pandemic phases and examined changes in feature importance.

## Patients and methods

### Patient collective and data

The study to generate the prediction model was approved by the Ethics Committee of the Medical Faculty of the Technical University of Munich (TUM) (253/19 S-SR, 11/06/2019), registered in Clinical Trials (NCT04092933, initial release 17/09/2019) and conducted at the University Hospital rechts der Isar of TUM. Informed consent was waived due to the retrospective nature of the study in accordance with German legal regulations. The study was performed in conformity with ethical guidelines, the Declaration of Helsinki and recommendations of the German Ethics Council. In accordance with legal data protection requirements, only de-identified data has been used.

The study was designed in concordance to the TRIPOD guidelines for reporting predictive model studies [7].

Data from all patients who underwent noncardiac surgery between June 2014 and October 2021 were included in the final analysis. Only the first surgery of each patient was of interest; subsequent surgeries were not considered further. Both elective and urgent procedures were included. Patients admitted to the ICU before the first surgery were excluded, as were patients who had a nonsurgical procedure (e.g., diagnostic) or an outpatient procedure.

**Fig. 1:**
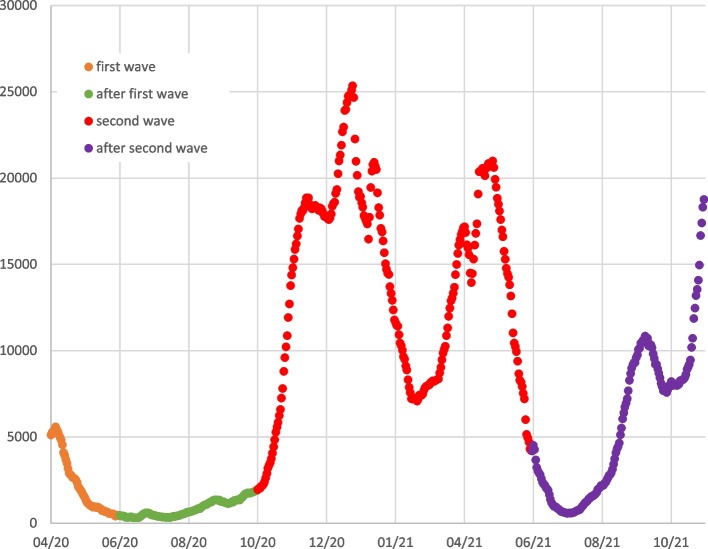
Pandemic course. Daily new infections, moving average over 7 days. Colour coded are the defined time periods of our study

**Fig. 2:**
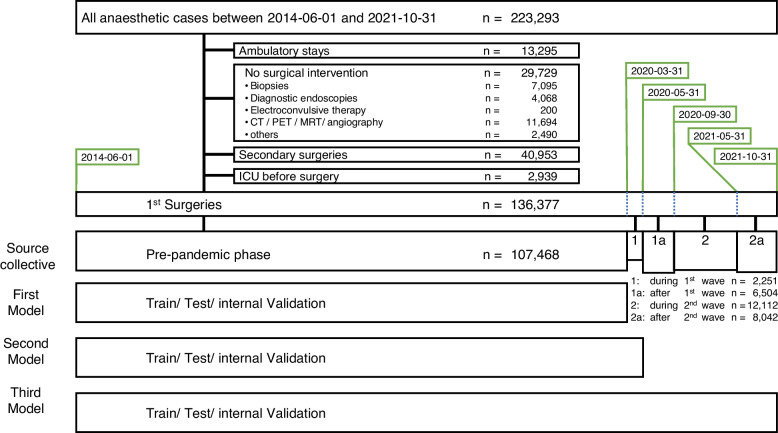
Strobe diagram. The area of the bar of “Source Collective” is proportional to the total number of patients in the given period, and the height is proportional to the number of patients per day

The data set was divided into different time periods: Patients treated before the Covid-19 pandemic (06/2014 – 03/2020), patients treated during the first pandemic wave (04/2020 – 05/2020), between the first and second pandemic waves (06/2020 – 09/2020), during the second pandemic wave (10/2020 – 05/2021), and after the second pandemic wave (06/2021 – 10/2021). Figure 1 shows the respective time period in context of the pandemic, Fig. 2 provides an overview of the patient numbers in each time period.

The dataset used included all available preoperative information from the hospital information system (SAP i.s.h.med), the laboratory information system (swisslab Lauris) and the anaesthesia patient data management system for the pre-anaesthesia visit (QCare, HIM-Health Information Management GmbH, Bad Homburg, Germany). Data that were not already available in tabular or coded form were structured using a quantity-based search algorithm, and drugs were assigned to their respective anatomical therapeutic chemical (ATC) code and summarized into groups, each with the same first four digits of the ATC code.

### Development of the XGBoost model

In total, we had over 12,000 parameters at our disposal, including 9300 surgical codes according to the German operation and procedure codes (OPS) and 780 laboratory values. Parameters such as medical history (241), movements within the hospital (24), medications (199), and preoperative orders from the blood depot (13) were also included in the final models. We did not impute missing values but created dichotomous variables about their availability and included this information in the model.

Models were trained and tested using datasets from three selected time periods. The datasets used for this purpose were stratified in a 3:1:1 ratio as a training cohort, a test cohort and an internal validation cohort. Randomization into training, test, and validation cohorts was performed in a way that the frequency of the mortality endpoint was the same in each cohort.

A total of three models were developed:

1. a model using the pre-pandemic dataset (06/2014—03/2020)
2. a model using the pre-pandemic data set and that of the first wave (06/2014—05/2020)
3. a model using data from the entire period (06/2014–10/2021).

After randomization, the proportion of patients from the first wave in training, testing, and validation cohorts of the second model was 2.0, 2.2, and 1.8%, respectively.

The predictive models were built using Extreme Gradient Boosting (XGBoost) with the following hyperparameters: “learning rate,” “minimum loss reduction,” “maximum depth of each tree,” “proportion of features,” “proportion of training samples,” “scale of positive weights,” and “minimum of instance weight” [8].

The limits of the hyperparameters were set as follows: Learning rate (0.01—0.2), minimum loss reduction (0—6), maximum depth of each tree (3—30 levels), proportion of features (0.5—1), proportion of training samples (0.5—1), scale of positive weights (0.01—10), and minimum sum of instance weights (0—20). Confidence intervals for each prediction were calculated using 100 bootstrap samples. Hyperparameter tuning was performed separately for each model using the Bayesian optimization method. After setting up the hyperparameter plane, 64 runs of parameter optimization were performed. The five best runs yielded very similar AUC values, ranging between 0.9329–0.9334, so the search was terminated at this point. Hyperparameter settings are provided in table A1 of supplementary file 1. After training, testing and internal validation, data from the different phases of the pandemic were used as external validation sets to compare the performance of the model in the different time periods. Evaluation plots of the XGBoost models are shown in figure F1 of supplementary file 1.

### Development of the deep learning model

With the same dataset and using the approximately 12,000 parameters mentioned above, Deep Learning (DL) neural networks were trained using the H2O framework in the R environment. An exhaustive grid search was performed using common hyperparameters such as learning rate, batch size, number of hidden layers, number of neurons per layer, activation and loss function, regularization and dropout rate. In this way, three DL models were created according to the time periods defined above using the same training, test and validation cohorts as for the XGBoost model.

### Statistical analysis

All analyses were performed using R, version 4.2.1 (R Foundation for Statistical Computing, Vienna, Austria). Models were compared based on their area under the receiver operating characteristic (AUROC) and area under precision-recall curve (AUPR) [95% confidence interval]. To further characterize the XGBoost models, a cut-off probability value for mortality was determined on the training sets using the Youden index. Based on this cut-off value, sensitivity, specificity, positive predictive value, and negative predictive value could be determined in each period to compare the performance of the models. Additionally, we calculated feature importance, i.e., the information gain of each feature as well as cover and frequency for each feature used in the XGBoost models.

## Results

### Patient characteristics and surgical spectrum

Patient characteristics in the different periods are shown in Table 1. The percentage of patients who died ranged from 0.8% before the pandemic to 1.0% during the second pandemic wave. Patients during and after the first and second wave were older than patients before the pandemic. During the pandemic, more patients fell into the American Society of Anaesthesiologists (ASA) 3 and 4 categories and were thus considered more severely ill overall. The number of emergencies was proportionally higher especially during the first wave. In terms of specialty departments, the proportion of patients in gynaecology/obstetrics and neurosurgery increased during the first wave of the pandemic. The frequency of surgeries performed outside regular operating hours (here from 08:00 to 18:00) and on weekends was highest during the first wave. Overall, changes were greatest during the first wave of the pandemic and partially normalized by the end of the study period. Median differences and percentage changes of the individual parameters in the pandemic waves compared with the pre-pandemic period are shown in table A2 of supplementary file 1.

**Table 1:** Patient characteristics and feature distribution in the respective timeframes

	**pre-pandemic**	**1**^**st**^** wave**	**after 1**^**st**^** wave**	**2**^**nd**^** wave**	**after 2**^**nd**^** wave**
**time period**	6/2014–3/2020	4/2020–5/2020	6/2020–9/2020	10/2020–5/2021	6/2021–10/2021
**number of cases**	107,468	2251	6504	12,112	8042
**mortality**	883 (0.8)	22 (1.0)	61 (0.9)	125 (1.0)	65 (0.8)
**age—years**	56 [38, 70]	58 [40, 72]	57 [39, 71]	57 [39, 72]	57 [38, 72]
**female sex**	49,282 (45.9)	1065 (47.3)	2896 (44.5)	5815 (48.0)	3789 (47.1)
**BMI missing**	34,276 (31.9)	484 (21.5)	1114 (17.1)	2061 (17.0)	1329 (16.5)
**BMI – kg/m**^2^	25.2 [22.5, 28.6]	24.9 [22.2, 28.4]	25.4 [22.6, 28.7]	25.3 [22.6, 28.7]	25.3 [22.5, 28.7]
**ASA missing**	31,407 (29.2)	440 (19.5)	1058 (16.3)	1917 (15.8)	1212 (15.1)
**ASA**					
** 1**	21,204 (27.9)	415 (22.9)	1392 (25.6)	2498 (24.5)	1637 (24.0)
** 2**	38,999 (51.3)	889 (49.1)	2667 (49.0)	5021 (49.2)	3417 (50.0)
** 3**	15,113 (19.9)	474 (26.2)	1317 (24.2)	2561 (25.1)	1700 (24.9)
** 4**	695 (0.9)	33 (1.8)	70 (1.3)	109 (1.1)	76 (1.1)
** 5**	50 (0.1)	0	0	6 (0.1)	0
** Mallampati available**	42,492 (39.5)	747 (33.2)	1634 (25.1)	2804 (23.2)	1624 (20.2)
**Mallampati**					
** I**	27,210 (41.9)	677 (45.0)	2358 (48.4)	4243 (45.6)	2906 (45.3)
** II**	28,633 (44.1)	603 (40.1)	1893 (38.9)	3824 (41.1)	2666 (41.5)
** III**	7242 (11.1)	177 (11.8)	495 (10.2)	1043 (11.2)	720 (11.2)
** IV**	1891 (2.9)	47 (3.1)	124 (2.5)	198 (2.1)	126 (2.0)
** count of preop consults**	2 [1, 3]	3 [2, 4.25]	3 [2, 4]	3 [2, 5]	3 [2, 5]
**department**					
** bone&joint**	19,153 (17.8)	334 (14.8)	1145 (17.6)	2037 (16.8)	1433 (17.8)
** gyn/obstetric**	10,692 (10.0)	293 (13.0)	647 (10.0)	1351 (11.2)	803 (10.0)
** head&neck**	25,171 (23.4)	402 (17.9)	1378 (21.2)	2406 (19.9)	1647 (20.5)
** neurosurgery**	10,259 (9.6)	315 (14.0)	720 (11.1)	1353 (11.2)	895 (11.1)
** outpatient**	9582 (8.9)	201 (8.9)	707 (10.9)	1347 (11.1)	880 (10.9)
** surgery**	20,397 (19.0)	421 (18.7)	1104 (17.0)	2087 (17.2)	1382 (17.2)
** urology**	12,160 (11.3)	285 (12.7)	801 (12.3)	1530 (12.6)	1002 (12.5)
**admission**					
** from external hospital**	2269 (2.1)	54 (2.4)	119 (1.8)	233 (1.9)	142 (1.8)
** child birth**	4236 (4.0)	103 (4.6)	258 (4.0)	496 (4.1)	327 (4.1)
** elective case**	77,843 (72.6)	1491 (66.8)	4704 (72.5)	8680 (71.9)	5689 (70.9)
** emergency**	19,342 (18.0)	509 (22.8)	1190 (18.3)	2306 (19.1)	1543 (19.2)
** new-born**	5 (0.0)	0	0	0	0
** other**	661 (0.6)	21 (0.9)	61 (0.9)	151 (1.3)	166 (2.1)
** polyclinic**	2853 (2.7)	54 (2.4)	154 (2.4)	213 (1.8)	159 (2.0)
** out-of-hour**	7810 (7.3)	204 (9.1)	480 (7.4)	887 (7.3)	577 (7.2)
** weekend**	4658 (4.3)	115 (5.1)	242 (3.7)	516 (4.3)	323 (4.0)
** PRCs ordered**	29,212 (34.3)	849 (41.7)	2163 (36.8)	4023 (36.5)	2292 (31.0)
** if yes: number**	4 [2, 4]	4 [2, 4]	4 [2, 4]	4 [2, 4]	2 [2, 4]
** FFPs ordered**	22,724 (26.7)	621 (30.5)	1590 (27.0)	2676 (24.2)	1301 (17.6)
** if yes: number**	4 [2, 4]	4 [2, 4]	4 [2, 4]	4 [2, 4]	4 [2, 4]
** PCC ordered**	441 (0.5)	12 (0.6)	40 (0.7)	91 (0.8)	47 (0.6)
** if yes: number**	4 [3, 4]	4 [4, 6]	4 [4, 6]	4 [3, 6]	4 [2, 4]
** CRP missing**	49,466 (46.0)	879 (39.0)	2419 (37.2)	4579 (37.8)	2937 (36.5)
** CRP—mg/L**	3.0 [1.0, 10.0]	3.0 [1.0, 16.0]	3.0 [1.0, 10.0]	3.0 [1.0, 10.0]	3.0 [1.0, 10.0]
** leukocytes missing**	25,031 (23.3)	267 (11.9)	734 (11.3)	1312 (10.8)	758 (9.4)
** leukocytes – 10**^**6**^**/µL**	7.3 [5.9, 9.1]	7.2 [5.8, 9.2]	7.2 [5.8, 9.0]	7.2 [5.8, 9.1]	7.2 [5.8, 9.1]
** albumin missing**	98,097 (91.3)	1976 (87.8)	5804 (89.2)	10,726 (88.6)	7168 (89.1)
** albumin—g/L**	44 [39, 46]	43 [38, 47]	44 [39, 47]	43 [37, 46]	43 [38, 46]
** Quick missing**	25,443 (23.7)	283 (12.6)	772 (11.9)	1372 (11.3)	794 (9.9)
** Quick value—%**	103 [95, 112]	116 [107, 120]	108 [99, 115]	107 [98, 114]	106 [98, 113]

Data are given as numbers (%) or median [interquartile range]. The laboratory values refer to those determined preoperatively

*BMI* Body-mass-index, *ASA* American Society of Anaesthesiologists Physical Score, *PRCs* Packed red cells, *FFPs* Fresh frozen plasma units, *PCCs* Platelet concentrates, *CRP* C-reactive protein

### XGBoost vs. deep learning neural network

For model comparison, we calculated both receiver operating characteristic (ROC) and precision-recall (PR) curves for each of the models, as the precision-recall-trade-off is a more suitable measure to determine model quality than AUROC in an imbalanced dataset [9].

**Fig. 3:**
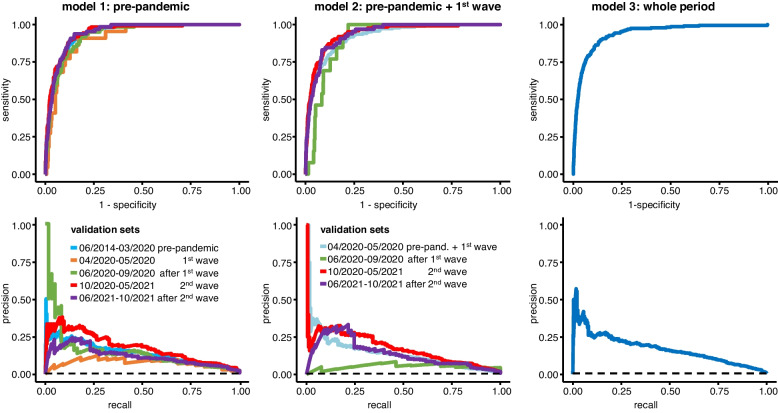
ROC- (first row) and PR-curves (second row) of the three XGBoost models. The dashed line shows the baseline mortality rate according to the performance of a random classifier

**Fig. 4:**
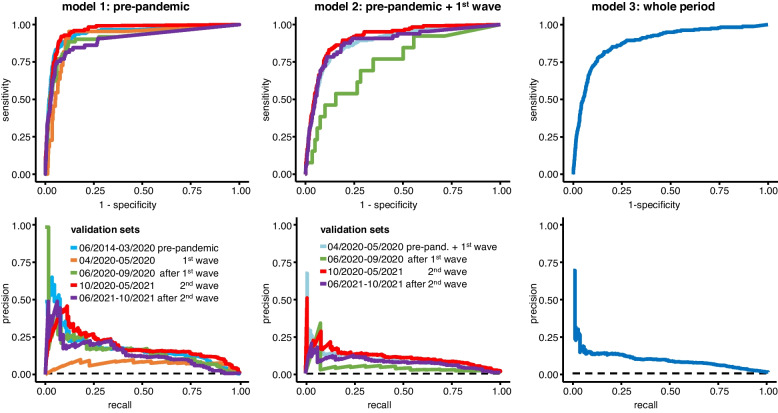
ROC- (first row) and PR-curves (second row) of the three DL models. The dashed line shows the baseline mortality rate according to the performance of a random classifier

XGBoost as well as the Deep Learning neural network (DL) show comparable AUROCs on the pre-pandemic data (0.951 [0.941–0.962] vs. 0.942 [0.921–0.962]). The precision-recall-trade-off is slightly better in DL. Both pre-pandemic models deteriorate when applied to first wave data in AUROC as well as in AUPR. The XGBoost model improves again in the post-wave one phases and shows stable performance overall, while DL improves, especially in terms of precision-recall trade-off, but continues to show fluctuations in AUROC. Similar results are observed for model two from pre-pandemic and first wave data. The XGBoost model trained on the entire data performs much better than the DL model. The performances of XGBoost and DL models are compared in Figs. 3 and 4 as well as in Table 2.

**Table 2:** Model performance measured by area under the receiver operating characteristic (AUROC-) and area under precision recall (AUPR-) curves [95% CI]

training set	validation parameter	validation set				
		**pre-pandemic**	**1**^**st**^** wave**	**after 1**^**st**^** wave**	**2**^**nd**^** wave**	**after 2**^**nd**^** wave**
		**06/2014–03/2020**	**04/2020–05/2020**	**06/2020–09/2020**	**10/2020–05/2021**	**06/2021–10/2021**
		**XGBoost**				
**pre-pandemic (model 1)**	AUROC	0.951 [0.941–0.962]	0.914 [0.871–0.957]	0.931 [0.909–0.953]	0.944 [0.929–0.959]	0.944 [0.927–0.961]
	AUPR	0.144 [0.140–0.149]	0.074 [0.064–0.086]	0.150 [0.141–0.159]	0.177 [0.171–0.184]	0.118 [0.111–0.125]
**pre-pandemic + 1**^**st**^** wave (model 2)**	AUROC	0.923 [0.907–0.940]		0.907 [0.870–0.943]	0.942 [0.924–0.959]	0.937 [0.917–0.958]
	AUPR	0.142 [0.138–0.147]		0.052 [0.041–0.066]	0.174 [0.169–0.179]	0.136 [0.129–0.144]
**whole set (model 3)**	AUROC	0.941 [0.927–0.954]				
	AUPR	0.168 [0.164–0.173]				
		**Deep Learning**				
**pre-pandemic (model 1)**	AUROC	0.942 [0.921–0.962]	0.914 [0.855–0.975]	0.907 [0.861–0.953]	0.958 [0.945–0.971]	0.899 [0.854–0.945]
	AUPR	0.187 [0.182–0.192]	0.074 [0.064–0.085]	0.160 [0.151–0.169]	0.193 [0.186–0.200]	0.145 [0.138–0.153]
**pre-pandemic + 1**^**st**^** wave (model 2)**	AUROC	0.877 [0.850–0.905]		0.747 [0.608–0.887]	0.912 [0.888–0.935]	0.884 [0.838–0.930]
	AUPR	0.080 [0.076–0.083]		0.041 [0.032–0.054]	0.106 [0.101–0.112]	0.073 [0.068–0.079]
**whole set (model 3)**	AUROC	0.885 [0.862–0.908]				
	AUPR	0.089 [0.085–0.092]				

### XGBoost feature importance

The XGBoost models, which show higher stability than the DL models in our study, are characterized in more detail below.

**Fig. 5:**
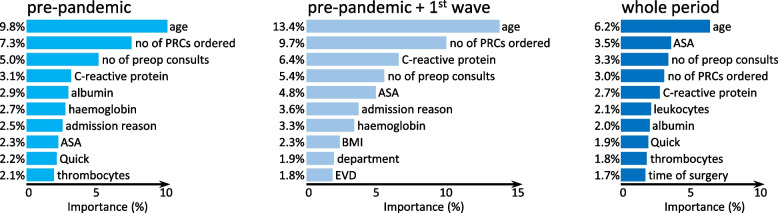
Importance of the top ten features of each model measured by the average gain of the feature if it is used in trees. PRCs = packed red cells, ASA = American Society of Anaesthesiologists Physical Score, EVD = external ventricular drain

In total, of the more than 12,000 possible features, 587 are used in the model from pre-pandemic data, 275 in the model from pre-pandemic and first wave data, and 923 in the model of the entire period. The most important features of each of the XGBoost models and their percentage share in the prediction are depicted in Fig. 5. In the pre-pandemic phase (model 1), age, number of packed red cells (PRCs) ordered and number of preoperative consults are the top three variables. The model including data from the first wave (model 2) shows an increasing importance of age and number of ordered packed red cells, whereas preoperative c-reactive protein (CRP) displaces the number of preoperative consults. In this model, the top three factors account for approximately 30% of the prediction. Throughout the period (model 3), age, ASA and number of preoperative consults are most important, however, individual importance decreases so that only age has an importance greater than 5%. Here, the three most important factors account for only 13% of the prediction. A table showing cover and frequency as well as the gain of all parameters used in the models are provided in supplementary file 2.

### XGBoost cut-off and performance metrics

Furthermore, we set a cut-off value based on the Youden-indices of the ROC curves of the three XGBoost models. In the first model, the threshold for predicting the death of a patient was set at a probability of 16.11, and in the second and third model, it was set at 18.05 and 12.96 respectively. At these thresholds, we determined the metrics of sensitivity, specificity, positive predictive value, and negative predictive value for each validation period. This consistently showed poor positive predictive value with acceptable sensitivity and good specificity while negative predictive value was consistently high. The specificity of the first model decreased significantly when applied to the first wave data. The other changes were not significant because of the wide confidence intervals. However, the second model is expected to lose sensitivity when applied to data from after the second wave. The cut-off values and metrics are shown in Table 3.

**Table 3:** Statistical assessment of different classifiers [95% CI] at the respective cut-off-values for the XGBoost models

training set	validation parameter	validation set					cut off from training set
		**pre-pandemic**	**1**^**st**^** wave**	**after 1**^**st**^** wave**	**2**^**nd**^** wave**	**after 2**^**nd**^** wave**	
		**06/2014–03/2020**	**04/2020–05/2020**	**06/2020–09/2020**	**10/2020–05/2021**	**06/2021–10/2021**	
**pre-pandemic (model 1)**	Sensitivity	0.722 [0.664–0.774]	0.773 [0.546–0.922]	0.672 [0.540–0.787]	0.760 [0.675–0.832]	0.677 [0.549–0.788]	0.1611
	Specificity	0.927 [0.924–0.930]	0.894 [0.880–0.906]	0.932 [0.925–0.938]	0.921 [0.916–0.926]	0.940 [0.935–0.946]	
	PPV	0.086 [0.075–0.098]	0.067 [0.039–0.105]	0.085 [0.062–0.114]	0.091 [0.074–0.110]	0.085 [0.062–0.112]	
	NPV	0.997 [0.996–0.998]	0.997 [0.994–0.999]	0.997 [0.995–0.998]	0.997 [0.996–0.998]	0.997 [0.996–0.998]	
	F1 Score	0.153 [0.135–0.1723]	0.123 [0.07–0.173]	0.151 [0.107–0.192]	0.163 [0.134–0.191]	0.151 [0.109–0.190]	
**pre-pandemic + 1**^**st**^** wave (model 2)**	Sensitivity	0.730 [0.660–0.792]		0.462 [0.192–0.749]	0.753 [0.685–0.812]	0.662 [0.534–0.774]	0.1805
	Specificity	0.915 [0.912–0.919]		0.927 [0.911–0.941]	0.920 [0.916–0.923]	0.933 [0.927–0.938]	
	PPV	0.065 [0.055–0.076]		0.061 [0.023–0.129]	0.082 [0.069–0.096]	0.074 [0.054–0.099]	
	NPV	0.998 [0.997–0.998]		0.994 [0.988–0.998]	0.997 [0.997–0.998]	0.997 [0.996–0.998]	
	F1 Score	0.119 [0.101–0.138]		0.108 [0.027–0.180]	0.148 [0.128–0.179]	0.134 [0.095–0.166]	
**whole set (model 3)**	Sensitivity	0.258 [0.204–0.319]					0.1296
	Specificity	0.992 [0.991–0.993]					
	PPV	0.221 [0.173–0.275]					
	NPV	0.994 [0.992–0.994]					
	F1 Score	0.238 [0.185–0.288]					

## Discussion

We developed machine-learning algorithms to predict perioperative mortality based on pre-operatively available data. Such models can aid decision-making during periods of scarce resources, like during the Covid-19 pandemic when intensive care beds for non-Covid patients were lacking. This makes the question of how robust such a model is to external influences all the more important. To address this issue, we developed three models with data from different phases before and during the pandemic using an XGBoost algorithm and a Deep Learning neural network. Our results show that precision-recall-trade-off was poor in both XGBoost and DL which is mostly due to an imbalanced data set: Mortality, as the end point of the study, is a very rare event with a frequency between 0.8 and 1.0%. AUPR decreases when the pre-pandemic models are used on first-wave data and recovers in the course. The same observation can be made after the first wave when the pre-pandemic and first wave data model is applied. In this respect, XGBoost and DL behave very similarly. The AUROC of the XGBoost models perform consistently very good with values > 0.9 while the DL model shows strong fluctuations in the individual pandemic phases, but both can recover fully or partially after an initial worsening.

To make the changes a little more descriptive, we have determined cut-off values for mortality prediction of the XGBoost models based on the Youden index. This illustrates that the proportion of false positive predictions is quite high, whereas the models perform very well by predicting negatives. It is evident that specificity and sensitivity show fluctuations in the different pandemic phases.

Gradient boosting methods are among the most commonly used algorithms in the field of perioperative medicine and often show excellent performance [10, 11]. However, there is evidence in the current literature that deep learning methods are superior to XGBoost with respect to AUROC [12] which made us use both methods. However, our results cannot support the superiority hypothesis for DL. Foremost, the DL models in our study showed much more pronounced fluctuations in AUROC than XGBoost, and our results show that the phenomenon of performance degradation under covariate shift is not limited to the XGBoost method.

It is generally assumed that different models react differently to changes. Overall, logistic regression models appear to be more vulnerable than machine learning algorithms [13]. Davis et al. studied the effects of a case mix shift on predictions and showed that neural networks are relatively robust, while random forests are moderately and most logistic regression models are strongly affected [14]. Unfortunately, XGBoost models, which are among the most frequently used algorithms for predictive models in perioperative medicine [10], were not examined in their study. However, from our data we can conclude that XGBoost as well as DL models may exhibit at least moderate susceptibility to covariate shift.

From our results it can be concluded that the restrictions of the first pandemic wave have a massive impact on model performance when taking into account that the patient population in this period is only a small fraction of the total population. This is caused by a change in patient characteristics and surgical spectrum with a shift towards urgent and emergency procedures which causes a covariate shift that affects model quality [15]. However, this problem is not new. For example, it is well known from economics that customer preferences change, making models based on old data inconsistent [16]. A change in the data on which the model is based can occur gradually, in the field of medicine, for example, as examination and treatment methods change over time. This can be addressed by removing older data from the data set or by applying factors so that old data is weighted weaker. Abrupt changes such as those caused by the Covid-19 pandemic, however, are more difficult to deal with and, although this problem seems obvious and might be clinically relevant, there is not much preliminary work so far on this issue.

The pandemic-related changes in the general conditions in our hospitals are manifold: Especially in the first phase of the Covid-19 pandemic, the surgical spectrum at our hospitals changed due to the rescheduling of elective procedures and a resulting proportional increase in emergency procedures with a significant decrease in hospital admissions and outpatient procedures. Case-mix-index and mortality rates increased [17]. There is evidence of worsening patient outcomes and a reduction in trauma cases during the first phase of the Covid pandemic [1, 18]. Less obvious changes involve a negative effect on the enrolment of patients in clinical trials [19] as well as a decrease in publications and scientific output of non-infectiology disciplines [20]. With the ongoing pandemic, conditions returned to normal [21]. As the pandemic progressed, delayed elective procedures that had accumulated, the so-called surgical back-log, had to be performed nonetheless, and so the numbers of surgeries normalized again [22].

These manifold dynamics which have the potential to cause significant covariate shift are reflected in our data. During the first wave, there were fewer elective cases, patients had higher ASA scores, and the surgical spectrum shifted toward departments that usually perform a greater proportion of urgent procedures, such as obstetrics or neurosurgery. In contrast to other reports, the number of out-of-hours surgeries in our institution increased, a fact that might also have contributed to poorer outcomes [23]. Looking at the figures after the second wave, they almost approached the pre-pandemic state again. Some changes remained, such as better documentation of presumed important information like the ASA score. Anaesthesiologists seemed to attach more importance to the ASA when the pandemic began, and documented this more frequently in the premedication protocol. This higher accuracy of documentation remained during the whole observation period.

Taken together, in the present study we face an abrupt onset of change in the data underlying the model which partially recedes after a period of several months.

Recently, Duckworth and colleagues developed an XGBoost model for prediction of hospital admission from the emergency department and examined data drift caused by the Covid-19 pandemic. In contrast to our study, they found a drop in AUROC after the onset of the pandemic whereas the AUPR increased [2]. However, these changes were caused by a shift in their target variable, admission rate, which increased markedly during the pandemic. In contrast, the target variable in our study, mortality, showed only fluctuations between 0.8 and 1.0%.

The study of Duckworth and colleagues also reports changes in feature importance. As an example, respiration rate rose in importance at the beginning of the lockdown and decreased during the course [2]. In our work we could observe similar phenomena. Regarding feature importance in our XGBoost models, the top variables are mostly the same, only in a different order. This is not surprising: age is an important variable in many models and scores for mortality prediction [24, 25]. The number of preoperative consults reflects a patient's comorbidities, which correlate with mortality just like the ASA score [26], while the number of blood products provided correlates with the severity of surgery. Only the importance of each variable and its place in the ranking of the top variables changes over the different phases of the pandemic.

Whether early re-training improves the predictive quality of the model remains a subject of discussion. There is some evidence that it might not be enough to just re-train the model with new data. Lacson and colleagues addressed the question whether re-training a model with new data will be sufficient or a newly developed model performs better. They came to the conclusion that a completely new developed model outperforms a model that was simply re-trained with augmented data [27]. We addressed this point by developing three different models for the respective periods using not only augmented data for updating but also performing hyperparameter optimization for each model.

Taken together, we can conclude that the performance of both DL and XGBoost models suffers due to shifts in the data. As a consequence, model performance has to be monitored to detect gradual as well as sudden data drift to regulate model updating cycles [3].

### Strengths and limitations

As a weakness could be considered that we provide models based on single-centre data with a relatively small number of patients in the first pandemic wave. However, at the onset of the Covid pandemic, patient numbers generally declined due to regulatory restrictions, and to our knowledge, no multicentre-generated models exist on this topic to date.

Furthermore, we chose mortality as a clearly defined endpoint that could easily be determined from routine data. As in-hospital death after surgery is a relatively rare event with a frequency of about 1%, this choice resulted in a highly imbalanced dataset. As a consequence, we received consistently good AUROCs but low precision-recall rates, and our models perform very well in predicting survivors at the price of a high false positive rate. However, it is precisely this weakness that illustrates the influence of data drift on performance metrics by causing a drastic decline in precision-recall trade-off with AUROCs being almost unaffected, at least in the XGBoost models.

Theoretically, the distribution shift in the data must be taken into account in model building, and appropriate techniques such as covariate shift adaptation should be used. We did not focus on this aspect in our work, because the onset of the Covid pandemic brought sudden unpredictable changes that were difficult to respond to in reality. The true extent of Covid-related changes in the patient and surgical spectrum in our hospitals is only now being analysed and published [28]. Any consideration of adjusting or controlling for the covariates therefore remains necessarily speculative. The fact that conditions in our case largely returned to normal after a few months was also not foreseeable at the beginning of the pandemic.

To date, there are few papers from the medical field that address the problem of sudden covariate shift [2]. Our work is intended to sensitize to this problem and supports the fact that further research is needed in this area.

## Conclusions

The present study has shown that a newly developed model with augmented data can perform worse under altered conditions after the initial phase of acute change. XGBoost models and Deep Learning neural networks are both susceptible to covariate shift, whereas XGBoost seems to be more stable in case of sudden changes, at least under the conditions we studied.

These findings tell us that updating a model too early can lead to a noticeable degradation in performance. Therefore, continued monitoring of a model’s predictive ability is necessary even after updating. A viable practical approach might be to use the old and updated models in parallel for a period of time after the update and compare their results. If the changes are only temporary, a model may regain its original predictive power.

## Supplementary Information

**Additional file 1: Table A1.** Hyperparameter settings of the XGBoost models. **Table A2.** Percentage changes / median differences of features in the different phases of the pandemic with 95% confidence intervals. **Figure F1.** The evaluation plots show the AUC (top) and logloss (bottom) as a function of the number of iterations.

**Additional file 2.**

## Data Availability

The dataset analysed during this study is not publicly available due to legal regulations. To gain access, proposals should be directed to the corresponding author. Requestors will need to sign a data access agreement.

## Abbreviations

ASA: American Society of Anaesthesiologists
AUROC: Area under receiver operating characteristic
AUPRC: Area under precision-recall curve
CRP: C-reactive protein
ICU: Intensive Care Unit
DL: Deep Learning neural network
PRC: Packed red cells
XGBoost: Extreme Gradient Boosting
