# Supplementary material for: Impact of the Covid-19 pandemic on the performance of machine learning algorithms for predicting perioperative mortality
Source: BMC Med Inform Decis Mak. 2023 Apr 12;23:67. doi: 10.1186/s12911-023-02151-1 (PMC10092913; doi:10.1186/s12911-023-02151-1)
Supplement: Supplementary file 1 — Additional file 1: Table A1. Hyperparameter settings of the XGBoost models. Table A2. Percentage changes / median differences of features in the different phases of the pandemic with 95% confidence intervals. Figure F1. The evaluation plots show the AUC (top) and logloss (bottom) as a function of the number of iterations. [file 12911_2023_2151_MOESM1_ESM.docx]

Additional tables and figures

|  | Learning rate | Gamma | Max depth | Min Child weight | Subsample | Colsample | Scale positive weight |
| --- | --- | --- | --- | --- | --- | --- | --- |
| pre-pandemic | 0.0194 | 2.65 | 5 | 0 | 0.673 | 0.641 | 13 |
| pre-pandemic + first wave | 0.0781 | 1.22 | 2 | 6 | 0.804 | 0.754 | 15.8 |
| whole period | 0.0198 | 1.2 | 12 | 1 | 0.64 | 0.59 | 1.92 |

Table A1: Hyperparameter settings of the XGBoost models

|  | **pre-pandemic vs. 1st wave** | **pre-pandemic vs. after 1st wave** | **pre-pandemic vs. 2nd wave** | **pre-pandemic vs. after 2nd wave** | **dimension** |
| --- | --- | --- | --- | --- | --- |
| **mortality** | -0.2 (-0.6 to 0.3) | -0.1 (-0.4 to 0.1) | -0.2 (-0.4 to -0.0) | 0.0 (-0.2 to 0.2) | percentage change |
| **age - years** | -2 [-4 to -1] | -1[ -2 to -1] | -1[ -2 to -1] | -1[ -2 to -1] | median difference |
| **female sex** | -1.5 (-3.6 to 0.7) | 1.3 (0.1 to 2.6) | -2.2 (-3.1 to -1.2) | -1.3 (-2.4 to -0.1) | percentage change |
| **BMI missing** | 10.4 (8.7 to 12.1) | 14.8 (13.8 to 15.7) | 14.9 (14.1 to 15.6) | 15.4 (14.5 to 16.2) | percentage change |
| **BMI – kg/m^2^** | 0.383 [0.086 to 0.553] | -0.129[ -0.267-0.000] | -0.018[ -0.155-0.075] | -0.018[ -0.173-0.106] | median difference |
| **ASA missing** | 9.7 (8.0 to 11.4) | 13.0 (12.0 to 13.9) | 13.4 (12.7 to 14.1) | 14.2 (13.3 to 15.0) | percentage change |
| **ASA** |  |  |  |  |  |
| **1** | 1.3 (-0.3 to 2.9) | -1.7 (-2.7 to -0.6) | -0.9 (-1.7 to -0.1) | -0.6 (-1.5 to 0.3) | percentage change |
| **2** | -3.2 (-5.3 to -1.1) | -4.7 (-6.0 to -3.5) | -5.2 (-6.1 to -4.2) | -6.2 (-7.3 to -5.1) | percentage change |
| **3** | -7.0 (-8.7 to -5.3) | -6.2 (-7.2 to -5.2) | -7.1 (-7.8 to -6.3) | -7.1 (-8.0 to -6.2) | percentage change |
| **4** | -0.8 (-1.3 to -0.3) | -0.4 (-0.7 to -0.2) | -0.3 (-0.4 to -0.1) | -0.3 (-0.5 to -0.1) | percentage change |
| **5** | 0.0 (0.0 to 0.1) | 0.0 (0.0 to 0.1) | -0.0 (-0.0 to 0.0) | 0.0 (0.0 to 0.1) | percentage change |
| **Mallampati available** | 6.4 (4.4 to 8.3) | 14.4 (13.3 to 15.5) | 16.4 (15.6 to 17.2) | 19.3 (18.4 to 20.3) | percentage change |
| **Mallampati** |  |  |  |  |  |
| **I** | -4.8 (-6.7 to -2.8) | -10.9 (-12.1 to -9.7) | -9.7 (-10.6 to -8.8) | -10.8 (-11.9 to -9.7) | percentage change |
| **II** | -0.1 (-2.0 to 1.7) | -2.5 (-3.6 to -1.3) | -4.9 (-5.8 to -4.1) | -6.5 (-7.6 to -5.4) | percentage change |
| **III** | -1.1 (-2.3 to 0.0) | -0.9 (-1.5 to -0.2) | -1.9 (-2.4 to -1.3) | -2.2 (-2.9 to -1.6) | percentage change |
| **IV** | -0.3 (-0.9 to 0.3) | -0.1 (-0.5 to 0.2) | 0.1 (-0.1 to 0.4) | 0.2 (-0.1 to 0.5) | percentage change |
| **count of preop consults** | -1 [-1 to -1] | -1[ -1--1] | -1[ -1--1] | -1[ -1--1] | median difference |

| **department** |  |  |  |  |  |
| --- | --- | --- | --- | --- | --- |
| **bone&joint** | 3.0 (1.5 to 4.5) | 0.2 (-0.7 to 1.2) | 1.0 (0.3 to 1.7) | 0.0 (-0.9 to 0.9) | percentage change |
| **gyn/obstetric** | -3.1 (-4.5 to -1.6) | 0.0 (-0.7 to 0.8) | -1.2 (-1.8 to -0.6) | -0.0 (-0.7 to 0.6) | percentage change |
| **head&neck** | 5.6 (3.9 to 7.2) | 2.2 (1.2 to 3.3) | 3.6 (2.8 to 4.3) | 2.9 (2.0 to 3.9) | percentage change |
| **neurosurgery** | -4.4 (-5.9 to -3.0) | -1.5 (-2.3 to -0.7) | -1.6 (-2.2 to -1.0) | -1.6 (-2.3 to -0.9) | percentage change |
| **outpatient** | -0.0 (-1.2 to 1.2) | -2.0 (-2.7 to -1.2) | -2.2 (-2.8 to -1.6) | -2.0 (-2.7 to -1.3) | percentage change |
| **surgery** | 0.3 (-1.4 to 1.9) | 2.0 (1.1 to 3.0) | 1.7 (1.0 to 2.5) | 1.8 (0.9 to 2.7) | percentage change |
| **urology** | -1.3 (-2.8 to 0.1) | -1.0 (-1.8 to -0.2) | -1.3 (-1.9 to -0.7) | -1.1 (-1.9 to -0.4) | percentage change |
| **admission** |  |  |  |  |  |
| **from external hospital** | -0.3 (-0.9 to 0.4) | 0.3 (-0.1 to 0.6) | 0.2 (-0.1 to 0.5) | 0.3 (0.0 to 0.7) | percentage change |
| **child birth** | -0.6 (-1.5 to 0.3) | -0.0 (-0.5 to 0.5) | -0.2 (-0.5 to 0.2) | -0.1 (-0.6 to 0.3) | percentage change |
| **elective case** | 6.2 (4.2 to 8.2) | 0.1 (-1.0 to 1.2) | 0.8 (-0.1 to 1.6) | 1.7 (0.7 to 2.7) | percentage change |
| **emergency** | -4.6 (-6.4 to -2.8) | -0.3 (-1.3 to 0.7) | -1.0 (-1.8 to -0.3) | -1.2 (-2.1 to -0.3) | percentage change |
| **new-born** | 0.0 (-0.0 to 0.0) | 0.0 (-0.0 to 0.0) | 0.0 (-0.0 to 0.0) | 0.0 (-0.0 to 0.0) | percentage change |
| **other** | -0.3 (-0.7 to 0.1) | -0.3 (-0.6 to -0.1) | -0.6 (-0.8 to -0.4) | -1.4 (-1.8 to -1.1) | percentage change |
| **polyclinic** | 0.3 (-0.4 to 0.9) | 0.3 (-0.1 to 0.7) | 0.9 (0.6 to 1.2) | 0.7 (0.4 to 1.0) | percentage change |
|  |  |  |  |  |  |
| **out-of-hour** | -1.8 (-3.0 to -0.6) | -0.1 (-0.8 to 0.5) | -0.1 (-0.5 to 0.4) | 0.1 (-0.5 to 0.7) | percentage change |
| **weekend** | -0.8 (-1.7 to 0.2) | 0.6 (0.1 to 1.1) | 0.1 (-0.3 to 0.5) | 0.3 (-0.1 to 0.8) | percentage change |
| **PRCs ordered** | -10.5 (-12.6 to -8.5) | -6.1 (-7.3 to -4.9) | -6.0 (-6.9 to -5.1) | -1.3 (-2.3 to -0.3) | percentage change |
| **if yes: number** | 0 [0 to 0] | 0[ 0-0] | 0[ 0-0] | 0[ 0-0] | median difference |
| **FFPs ordered** | -6.4 (-8.3 to -4.6) | -3.3 (-4.4 to -2.2) | -0.9 (-1.7 to -0.2) | 5.0 (4.1 to 5.8) | percentage change |
| **if yes: number** | 0 [0 to 0] | 0[ 0-0] | 0[ 0-0] | 0[ 0-2] | median difference |
| **PCC ordered** | -0.1 (-0.4 to 0.2) | -0.2 (-0.4 to 0.0) | -0.3 (-0.5 to -0.2) | -0.2 (-0.4 to 0.0) | percentage change |
| **if yes: number** | 0 [-2 to 0] | 0[ 0-0] | 0[ 0-0] | 0[ 0-0] | median difference |
| **CRP missing** | 7.0 (4.9 to 9.0) | 8.8 (7.6 to 10.1) | 8.2 (7.3 to 9.1) | 9.5 (8.4 to 10.6) | percentage change |
| **CRP - mg/L** | 0 [-0.1 to 0] | 0[ 0-0] | 0[ 0-0] | 0[ 0-0] | median difference |
| **leukocytes missing** | 11.4 (10.0 to 12.8) | 12.0 (11.2 to 12.8) | 12.5 (11.8 to 13.1) | 13.9 (13.2 to 14.6) | percentage change |
| **leukocytes – 10^6^/µL** | 0.020 [-0.125 to 0.180] | 0.100[ 0.010-0.200] | 0.070[ 0.000-0.120] | 0.090[ 0.000-0.170] | median difference |
| **albumin missing** | 3.5 (2.1 to 4.9) | 2.0 (1.3 to 2.8) | 2.7 (2.1 to 3.3) | 2.1 (1.4 to 2.9) | percentage change |
| **albumin - g/L** | 0.100 [-0.100 to 0.200] | 0.000[ -0.100-0.100] | 0.100[ 0.000-0.150] | 0.100[ 0.000-0.200] | median difference |
| **Quick missimg** | 11.1 (9.7 to 12.5) | 11.8 (11.0 to 12.6) | 12.3 (11.7 to 13.0) | 13.8 (13.1 to 14.5) | percentage change |
| **Quick value - %** | -13 [-14 to -13] | -5[ -5--4] | -4[ -4--3] | -3[ -3--3] | median difference |

Table A2: Percentage changes / median differences of features in the different phases of the pandemic with 95% confidence intervals

Figure F1: The evaluation plots show the AUC (top) and logloss (bottom) as a function of the number of iterations.
